# Sol-Gel Derived Silica-Titania Waveguide Films for Applications in Evanescent Wave Sensors—Comprehensive Study

**DOI:** 10.3390/ma15217641

**Published:** 2022-10-31

**Authors:** Paweł Karasiński, Magdalena Zięba, Ewa Gondek, Jacek Nizioł, Sandeep Gorantla, Krzysztof Rola, Alicja Bachmatiuk, Cuma Tyszkiewicz

**Affiliations:** 1 Department of Optoelectronics, Silesian University of Technology, ul. B. Krzywoustego 2, 44-100 Gliwice, Poland; 2 Institute of Physics, Cracow University of Technology, ul. Podchorążych 1, 30-084 Kraków, Poland; 3 Faculty of Physics and Applied Computer Science, AGH University of Science and Technology, al. Mickiewicza 30, 30-059 Krakow, Poland; 4 Łukasiewicz Research Network—PORT Polish Center for Technology Development, ul. Stabłowicka 147, 54-066 Wrocław, Poland

**Keywords:** integrated optics, light scattering, optical losses, planar waveguide, sol gel, silica-titania, dip-coating

## Abstract

Composite silica-titania waveguide films of refractive index ca. 1.8 are fabricated on glass substrates using a sol-gel method and dip-coating technique. Tetraethyl orthosilicate and tetraethyl orthotitanate with molar ratio 1:1 are precursors. Fabricated waveguides are annealed at 500 °C for 60 min. Their optical properties are studied using ellipsometry and UV-Vis spectrophotometry. Optical losses are determined using the streak method. The material structure and chemical composition, of the silica-titania films are analyzed using transmission electron microscopy (TEM) and electron dispersive spectroscopy (EDS), respectively. The surface morphology was investigated using atomic force microscopy (AFM) and scanning electron microscopy (SEM) methods. The results presented in this work show that the waveguide films are amorphous, and their parameters are stable for over a 13 years. The optical losses depend on their thickness and light polarization. Their lowest values are less than 0.06 dB cm^−1^. The paper presents the results of theoretical analysis of scattering losses on nanocrystals and pores in the bulk and interfaces of the waveguide film. These results combined with experimental data clearly indicate that light scattering at the interface to a glass substrate is the main source of optical losses. Presented waveguide films are suitable for application in evanescent wave sensors.

## 1. Introduction

Integrated optics dates back to the second half of the 1960s when it was created as a new field of scientific activity based on waveguide optics and thin-film technologies [1,2]. Owing to the use of thin-film technologies, we can fabricate many optical elements on a common substrate, integrated with each other into functional systems. Through the integration of many optical elements on one substrate, we can avoid problems related to their mutual alignment. Moreover, such systems are resistant to vibrations, and the applied technological procedures guarantee a high degree of repeatability of the parameters of the fabricated structures. There are two main stages in the technological processes of integrated optics. In the first stage, waveguide layers are produced, and in the second stage planar optical systems are created in subsequent technological processes. These technological processes generally consist in selective masking and then etching of the waveguide layer followed by the epitaxy of successive layers with the desired properties. Waveguide layers are material platforms based on which integrated optics technologies are developed. Currently, the main areas of application of integrated optics are: data centers [3,4], optical telecommunication [5,6,7,8,9], and evanescent wave sensors [10,11,12,13,14,15,16]. The emerging area of application for integrated optics involves optical computing [17], and quantum photonics [18,19].

Integrated optics systems for applications in data centers, telecommunications, optical computing and quantum photonics are designed for the NIR spectral range and they are developed on the basis of two mature material platforms: silica [7,8,20,21,22], and indium phosphide [3,4,5,6,7,8,9,17,18,19]. By combining both material platforms having high refractive indexes, using CMOS (complementary metal oxide semiconductor) processes, we can fabricate fully functional processing systems of optical signals with high integration scale [6,23]. Another material platform for applications in the Vis-NIR spectral range, including sensor applications in the Vis range, is silicon nitride Si_3_N_4_ [15,24]. On the basis of Si_3_N_4_ and SiO_2_ the TriPlex material platform was developed [25,26]. A review of low-cost techniques for producing waveguide films is presented in Ref. [27]. The development of a technology for the fabrication of waveguide layers that can be accepted as a material platform for integrated optics is a difficult technological challenge, whereof implementation entails long and costly research. The difficulties in developing material platforms result from the requirements that they have to meet. These requirements include high refractive index, low optical loss, long-term stability of parameters and high chemical resistance, which is particularly important for the chemical/biochemical evanescent wave sensors technology. High refractive index of the waveguide layer determines the attainment of a large integration scale of integrated optics systems, and in the case of planar chemical/biochemical evanescent wave sensors, it is indispensable to achieve high optical sensitivity [28,29]. The problem of high scattering losses resulting from the roughness of waveguide layer surface was faced as early as at the beginning of the development process of integrated optics [2]. This problem is still existing nowadays, and it is one of the issues analyzed in this work. A particularly difficult technological challenge is to obtain low optical losses with high refractive index of the waveguide layer and with its thickness slightly greater than the cut-off thickness. The impact of waveguide layer parameters on optical losses is analyzed in detail in Section 2.

The concepts of many basic elements of integrated optics were developed and preliminarily verified as early as at the beginning of the 1970s [2,30]. However, the development of integrated optics was hampered for many years by the lack of appropriate material platforms, i.e., homogeneous waveguide layers with high refractive indexes and low optical losses. At that time, an important role in the development of integrated optics was played by gradient-index waveguides fabricated by using ion exchange in glass [31,32], and metal-diffused optical waveguides in lithium niobate (LiNbO_3_) [33,34,35]. These technologies contributed to the development of measurement methods as well as analysis and design methods of integrated optics systems. However, the disadvantage of gradient-index waveguides lies in their incompatibility with telecommunications optical fibers and in low contrast of refractive index. The gradient profile of the refractive index of waveguides adversely affects optical sensitivities of the evanescent wave sensors [36]. Lithium niobate waveguides LiNbO_3_ compensate all aforementioned deficiencies with excellent electro-optical properties, which make them still applicable in the production of active integrated optics systems [35]. The developed in the past decades “ion-cut” technique is also used for the fabrication of sub-micrometer thickness crystalline LiNbO_3_ thin films. This technique has opened up new possibilities for the use of lithium niobate in integrated optics technology [37].

A breakthrough in the development of integrated optics started with the growing interest in it by optical telecommunications sector, which contributed to the development of the currently dominant material platforms Si and InP [3,7,8,9,17,18,19,20,21,22]. Silica with the refractive index ca. 3.5 for the wavelength of 1550 nm offers the potential to fabricate integrated optics systems with a very high scale of integration. In addition, the use of silicon waveguide layers in evanescent wave sensors additionally offers the possibility to obtain extremely high optical sensitivities. However, silicon is prone to oxidation, it is not transparent in the Vis range and the costs of technological equipment are very high. Hence, the silicon platform is not attractive to small and medium-sized enterprises. For this reason, many research groups are trying to develop other, low-cost material platforms for applications in integrated optics technology [38,39,40,41], including chemical/biochemical evanescent wave sensors, which can be used in the Vis-NIR spectral range. Such research was also undertaken by our research group, and the results are presented in this article.

Very high chemical resistance is exhibited by transition metal oxides (ZnO, TiO_2_, HfO_2_, ZrO_2_), which are potentially suitable materials for waveguide layers for planar evanescent wave chemical/biochemical sensors. The highest refractive index among transition metal oxides is displayed by TiO_2_. Since the maximum optical sensitivities in evanescent wave sensors are proportional to the refractive index of a waveguide layer, it means that TiO_2_ waveguide layers offer potentially the highest optical sensitivities. However, TiO_2_, similar to other transition metal oxides, tends to crystallize and to form a polycrystalline structure, which in the case of waveguide layers results in a drastic increase in scattering losses due to the development of nanocrystals and the rise of surface roughness. The crystallization of TiO_2_ can be weakened by adding silica dioxide [42,43,44,45]. Thus, using binary oxides SiO_2_:TiO_2_ systems, it is possible to produce layers with refractive indexes ranging from 1.45 to 2.55 at visible and near-infrared wavelength [44]. The structure of the SiO_2_:TiO_2_ composite layer depends on its chemical composition and annealing temperature. Yet, it should be emphasized that the higher the content of TiO_2_ in the SiO_2_:TiO_2_ composite material, the more difficult it is to avoid the crystallization of TiO_2_, i.e., to obtain an amorphous structure. Probably for this reason, while developing composite SiO_2_:TiO_2_ waveguide layers, many researchers restricted themselves to the fabrication of compositions with titanium content below wt. 20%. The crystallization of TiO_2_ in the composite layer leads to the formation of a TiO_2_ phase separated from the SiO_2_ phase, which results in strong light scattering.

A suitable fabrication method of binary SiO_2_:TiO_2_ composite systems is the sol-gel method [46], which does not require expensive technological equipment, it is efficient and allows for the production of waveguide layers with surface smoothness at the molecular level [47]. Composite binary oxides SiO_2_:TiO_2_ waveguide films produced by sol-gel and dip-coating techniques are the subject of this article. Silica-titania waveguide films have been already presented in many scientific articles [48,49,50,51,52,53,54,55,56,57,58,59,60,61,62,63]. For the first time silica-titania waveguide films were fabricated in 1983 via sol-gel and dip coating technique by Herrmann and Wildmann [48]. Further, the development of sol-gel based silica-titania films was significantly contributed by the group led by Tiefenthaler [49]. In both cases the same Liquidcoat solution supplied by E. MERCK was used (Si:Ti = 1). Based on the optical waveguide layers produced in this way, many chemical and biochemical evanescent wave sensors were developed in the group led by Lukosz [49,50,51,52]. Silica-titania waveguide films with the molar ratio of Si:Ti~1 have been also reported by many other research groups [53,54,55,56,57], as well as by the authors of this work [28,58,59,60,61,62,63]. Tiefenthaler and Lukosz in the research on silica-titania waveguide films with the molar ratio of Si:Ti = 1 and refractive index of *n*_1_~1.72 measured optical losses of ~0.6 dB cm^−1^ for the wavelength *λ* = 632.8 nm [49]. Weisenbach and Zelinski in the research on composite waveguide layers SiO_2_:TiO_2_ (Si:Ti = 1) with the refractive index *n*_1_ = 1.75 and thickness *d* = 180 nm determined the optical losses equal to 1.4 dB cm^−1^, which after 5 months of storage of the structures increased to 4.2 dB cm^−1^ [53]. Presumably, so far it is the only case, apart from our previous work [61], where the results of research on the temporal stability of waveguide layers produced by the sol-gel method were reported. Jiwei et al. reported composite binary oxides SiO_2_:TiO_2_ waveguide layers produced by the sol-gel method with the refractive index *n*_1_ = 1.83 (Si:Ti~1) for which they measured the optical loss equal to 7.4 dB cm^−1^ [55]. We demonstrated that the composite waveguide layers SiO_2_:TiO_2_ developed by us have low optical losses (<0.2 dB cm^−1^), which remain unchanged for 5 years [61]. In this work, we show that the waveguide layers we have developed retain their properties over a period of 13 years. In the paper published later we demonstrated that the waveguide layers developed by us are chemically and optically homogeneous and the obtained silica and titania oxides are not stoichiometric [63]. For this reason, in the further part of this work we will be referring to our layers as SiO_x_:TiO_y_ composite waveguide layers. One of our earlier papers concerns the fabrication of grating couplers and analysis of their sensor properties [59], while three other concern rib waveguides and directional couplers fabricated with the use of the traditional optical contact photolithography and wet chemical etching [58,60,62]. Here, we present for the first time the results of a comprehensive study on the SiO_x_:TiO_y_ composite waveguide layers developed by us. Our motivation to undertake research presented here was driven by the necessity to develop a low-cost material platform for the development of planar chemical/biochemical evanescent wave sensors.

In this article, we present the results of research on optical, chemical and structural properties of the material. Special attention was paid to propagation losses. The SiO_x_:TiO_y_ composite waveguide layers presented in this work are made with the sol-gel method and dip-coating technique, and hence we present relationships binding the substrate withdrawal speed and final thicknesses and refractive indexes of fabricated films. The dispersion characteristics of the refractive index *n* and extinction coefficient *κ* were determined by spectroscopic ellipsometry. Optical homogeneity of the produced SiO_x_:TiO_y_ composite layers was tested by reflection spectrophotometry. The positions of the absorption edges were determined using transmission spectrophotometry. Using the Tauc method, the widths of optical band gaps were determined. In the studies on the morphology of the surface of the layers and glass substrates, the atomic force microscopy (AFM) and scanning electron microscopy (SEM) methods were used. The tests with the use of transmission electron microscopy (TEM) confirmed the amorphous nature of our layers. Waveguide properties and propagation losses were investigated using the *m*-line method. To interpret the results of propagation losses, theoretical analyses were performed. Optical losses measured by the streak method are at the level of the calculated scattering losses at the interfaces of the waveguide layer. We present experimental evidence confirming that the waveguide layers we have developed remain stable over many years. There is an excellent agreement between the test results obtained with the use of different measurement methods. The measured optical losses agree reasonably well with the theoretical predictions.

The organization of the work is as follows. Section 2 presents the optical loss model in the optical waveguide. We present the results of theoretical analysis involving the impact of waveguide layer thickness, roughness and autocorrelation length on optical scattering losses. Section 3 contains the technological procedure for the fabrication of composite waveguide layers SiO_x_:TiO_y_, and Section 4 comprises the applied methods and measuring equipment. Section 5 contains the research results and their discussion. Finally, the results presented in this work are summarized in Section 6.

## 2. Optical Loss—Theoretical Background

**Figure 1:**
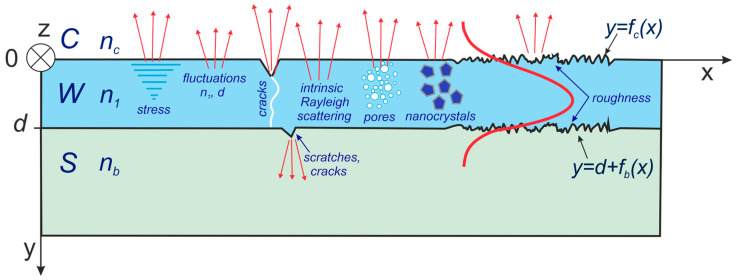
Scheme of the slab waveguide and sources of loss. S—substrate, W—waveguide film, C—cover, *n_b_*, *n_1_*, *n_c_*—refractive indexes of substrate, waveguide film and cover, respectively.

The paper presents three-layer waveguide structures whereof diagram is presented in Figure 1. On the substrate S with the refractive index *n_b_* a waveguide layer W with the refractive index *n*_1_ was applied, whereof cover C has the refractive index *n_c_*. The characteristic equations of such a waveguide structure are as follows:

(1) $$\tan^{-1}\left[{\left(\frac{n_{1}}{n_{b}}\right)}^{\rho }\sqrt{\frac{N^{2}-n_{b}^{2}}{n_{1}^{2}-N^{2}}}\right]+\tan^{-1}\left[{\left(\frac{n_{1}}{n_{c}}\right)}^{\rho }\sqrt{\frac{N^{2}-n_{c}^{2}}{n_{1}^{2}-N^{2}}}\right]=\frac{2\pi }{\lambda }d\sqrt{n_{1}^{2}-N^{2}}+m\pi$$

where *ρ* = 0 and 1 for the polarization TE and TM, respectively.

Optical losses in a slab waveguide are effected by the absorption and scattering of light. Absorption losses occur mainly in the vicinity of the absorption edge and, in the case of doping with active ions, also within the absorption bands of the introduced dopants. The absorption edges are generally in the UV spectral range. The position of the absorption edge as well as that of the absorption bands can be easily determined from spectrophotometric measurements.

The paper presents two-component composite waveguide layers SiO_x_:TiO_y_ fabricated by the sol-gel method and dip-coating technique. Potential sources of scattering losses that may occur in these layers are shown in Figure 1. Composite waveguide layers are produced from the liquid phase. The final layer thickness may be even 10 times smaller than the thickness of the sol layer immediately after its application on the substrate. Hence, there is a high probability of large stresses occurring in the waveguide layer after drying and annealing. These stresses can lead to the development of non-homogeneity of the refractive index and to thickness fluctuations, or it can even cause cracking of layers. Non-homogeneities of the refractive index can be detected by spectrophotometric measurements (Section 5.2). The SiO_x_:TiO_y_ composite waveguide layers produced by the sol-gel method are characterized by the presence of residual porosity as their natural feature. In binary SiO_x_:TiO_y_ waveguide layers, when the content of titanium dioxide exceeds wt. 20%, it tends to crystallize and to form a separated phase, which may result in cracking of the layers in the annealing processes or even their delamination from the substrate. With the rise of the annealing temperature, residual porosity decreases, and nanocrystals grow up. The presence of nanocrystals also negatively affects the smoothness of the surface of the waveguide layer. Avoiding or reducing these undesirable effects is a difficult technological challenge. The surfaces of the applied glass substrates may contain scratches or gouged out spots. Such substrate defects, cracks in the waveguide layer or scratches will be the source of Mie scattering. Such defects practically disqualify optical waveguide layer. The presence of such defects can be easily determined by its visual observation, or by excitation in the Vis spectral range. These defects can be relatively easily eliminated by improving technological processes, and then they should not occur in good quality waveguide layers.

Inhomogeneity of material at the molecular level, the presence of pores and nanocrystals as well as natural roughness of the substrate surface or waveguide layer surface are the source of light scattering, and they are accountable for optical losses in the waveguide layer. Light scattering on these inhomogeneities has the character of Rayleigh scattering. Thus, the optical losses in the waveguide layer can be written as:

(2) $$\alpha =\alpha_{Ri}+\alpha_{p}+\alpha_{n-c}+\alpha_{r}$$

where: *α_Ri_*—intrinsic material scattering loss, *α_p_*—pores loss, *α_n−c_*—nanocrystal loss, *α_r_*—roughness loss. The scattering losses in the SiO_2_:TiO_2_ composite material (Si:Ti = 0.8:0.2) were analyzed in the work [64]. The analysis of intrinsic material scattering loss using the model of Pinnow et al. showed that these losses are practically independent of the content of titania, and they are at the level ca. 1.0 × 10^−3^ dB cm^−1^ [65]. Such losses are much smaller than other losses, and we consider them negligible in the further part of this work.

The problem of optical loss due the roughness scattering was intensively investigated in the past. Tien, using a simple specular reflection model in a slab waveguide structure and using the Rayleigh criterion, derived the formula for scattering losses of the modes guided in it [66]. Marcuse advanced the coupled-mode theory and developed a determination method of losses based on the coupling analysis between the guided modes and radiation modes [67,68]. Ames and Hall derived the modal attenuation coefficients by means of the first-order boundary perturbation theory [69]. Lacey and Payne, using perturbation theory and treating the waveguide as a radiating antenna, with the random wall imperfections as an equivalent current source, derived the modal attenuation coefficient for a slab asymmetrical waveguide [70,71]. The 2-D Lacey and Payne formalism is often applied to 3-D rectangular waveguides, where the 3-D structure is replaced by a 2-D one using effective index method [72,73,74,75]. In this work, we use 2-D Lacey and Payne formalism to analyze scattering losses in the sol-gel and dip-coating silica-titania slab waveguides that we fabricate. Later in this section, we present the Lacy-Payne method of analysis of scattering losses at the interfaces of the waveguide layer and the Rayleigh-Mie scattering theory on nanocrystals.

### 2.1. Model of the Interface Scattering Losses

In the theoretical analysis, we examine scattering losses occurring on the surfaces delimiting the waveguide layer. For this purpose, we use the Lacey-Payne model [69,71]. The interface surfaces of the waveguide layer shown in Figure 1 are rough and their profiles are described respectively by the function *f_c_*(*x*) at the interface with the cover C and *f_b_*(*x*) at the interface with the substrate S. Accordingly with Lacey-Payne model, the exponential radiation loss coefficient for scattering by surface roughness in single mode waveguide is given as [70,71]:

(3) $$\mu_{\xi }=\Phi_{\xi }^{2}\cdot {\left(n_{1}^{2}-n_{\xi }^{2}\right)}^{2}\frac{k_{0}^{3}}{8\pi n_{1}}\int_{0}^{\pi }\tilde{R}\left(\beta -n_{\xi }k_{0}\cos\theta \right)d\theta$$

This attenuation coefficient *μ_ξ_* is defined by the relation: $P(x)=P(0)\cdot \exp(-\mu_{\xi }x)$, where *P*(*x*) is an optical power in a point *x*, *ξ* = *b*,*c* (Figure 1). In Equation (3) *β* = *k*_0_*N* is a propagation constant, where *k*_0_ = 2*π*/*λ* is a wave number in free space, corresponding with wavelength *λ.* Quantity $\Phi_{\xi }$ is the amplitude of modal field at the interface *y* = *d* (subscript *ξ* = *b*) and *y* = 0 (subscript *ξ* = *c*) respectively. Amplitude of modal field fulfils the condition:

(4) $$\int_{-\infty }^{+\infty }\Phi^{2}(y)dy=1$$

When this condition is met, the square of the modal amplitude *Φ*^2^(*y*) is the optical power density in the *y*-plane. The integral function ${\tilde{R}}_{\xi }(\Omega_{\xi })$ in Equation (3) is a spectral density function:

(5) $${\tilde{R}}_{\xi }\left(\Omega_{\xi }\right)=\int_{-\infty }^{+\infty }R_{\xi }(u)\exp(i\Omega_{\xi }u)du$$

*R_ξ_*(*u*) is an autocorrelation function of a profile *f_ξ_*(*x*), defined as:

(6) $$R_{\xi }(u)=\underset{L\to \infty }{\lim}\frac{1}{2L}\int_{-L}^{+L}f_{\xi }(x)f_{\xi }(x+u)dx$$

For most engineering surfaces the exponential form of the autocorrelation *R_ξ_*(*u*) is used and can be written as:

(7) $$R_{\xi }(u)=\sigma_{\xi }^{2}\exp\left(-\frac{\left|u\right|}{L_{c,\xi }}\right)$$

where *L_c,__ξ_* is equal to the autocorrelation length, typically defined as the distance at which the value of the autocorrelation function is 1/*e* of its original value. *σ_ξ_* is the standard deviation of the ordinate distribution. In some cases, the autocorrelation function may take the form of a Gaussian distribution:

(8) $$R_{\xi }(u)=\sigma_{\xi }^{2}\exp\left(-\frac{u^{2}}{L_{c,\xi }^{2}}\right)$$

The analysis of AFM (atomic force microscopy) images of the surface of our waveguide layers and the applied glass substrates shows that the corresponding autocorrelation functions have exponential character. For exponential autocorrelation function Equation (3) takes the form [71]:

(9) $$\mu_{\xi }=\frac{\sqrt{2}}{2}\Phi_{\xi }^{2}\Delta_{\xi }^{2}n_{1}^{3}k_{0}^{3}\sigma_{\xi }^{2}L_{c,\xi }\Gamma \left(\beta ,L_{c,\xi }\right),$$

where: $\Delta_{\xi }=\left(n_{1}^{2}-n_{\xi }^{2}\right)/\left(2n_{1}^{2}\right)$ is a contrast of the refractive index, and,

(10) $$\Gamma \left(\beta ,L_{c,\xi }\right)={\left[\frac{{\left(4\beta^{2}L_{c,\xi }^{2}+Q_{\xi }^{2}\right)}^{1/2}+Q_{\xi }}{4\beta^{2}L_{c,\xi }^{2}+Q_{\xi }^{2}}\right]}^{1/2},$$

whereas $Q_{\xi }=1-L_{c,\xi }^{2}\gamma_{\xi }^{2}$, and $\gamma_{\xi }^{2}=\beta^{2}-n_{\xi }^{2}k_{0}^{2}$. The optical loss (in dB⋅cm^−1^) is calculated as $\alpha_{\xi }=4.343\mu_{\xi }$. It can be seen from the Equation (9) that the optical scattering losses on the interface unevenness of the waveguide layer clearly depend on optical power density on boundary surfaces of the waveguide layer, the squared contrast of the refractive index, refractive index of the waveguide layer in the third power, wavenumber in free space well as on the squared roughness of the surface and on the autocorrelation length. The dependence of the last factor *Γ* on the length of the autocorrelation and on the thickness of the waveguide layer will be demonstrated later in the paper. The computational results illustrating the influence of the parameters of the structure on the scattering losses at the interfaces of the waveguide layer are presented in Section 2.3.

### 2.2. Rayleigh-Mie Theory

We calculated the scattering losses effected by nanocrystals and residual porosity using Rayleigh-Mie theory, with the application of dependences given in the paper [64]. This model assumes light scattering on spherical, non-absorbent particles of the same dimensions, whereof diameters are much smaller than the wavelength. These particles can be nanocrystals or pores of nanometer size. The exponential radiation loss coefficient is given as follows [64]:

(11) $$\mu_{p,n-c}=389.6\cdot {\left(\frac{{\left(n_{2}/n_{1}\right)}^{2}-1}{{\left(n_{2}/n_{1}\right)}^{2}+2}\right)}^{2}f_{p,n-c}\cdot \frac{D_{{}_{p,n-c}}^{3}}{\lambda^{4}},\;$$

where $D_{p,n-c}$ is the diameter of pores/nanocrystals, $f_{p,n-c}$ is the fraction of the material volume occupied by the pores/nanocrystals, *n*_2_ is the refractive index in the volume of pores/nanocrystals, *n*_1_ is the refractive index of the matrix in which the pores/nanocrystals are suspended. The optical loss (in dB⋅cm^−1^) is calculated as follows:

(12) $$\alpha_{p,n-c}=4.343\mu_{p,n-c}\int_{0}^{d}G(y)dy,$$

where $G\left(y\right)\equiv \Phi^{2}\left(y\right)$ is a normalized optical power density.

### 2.3. Optical Loss—Calculation Results

**Figure 2:**
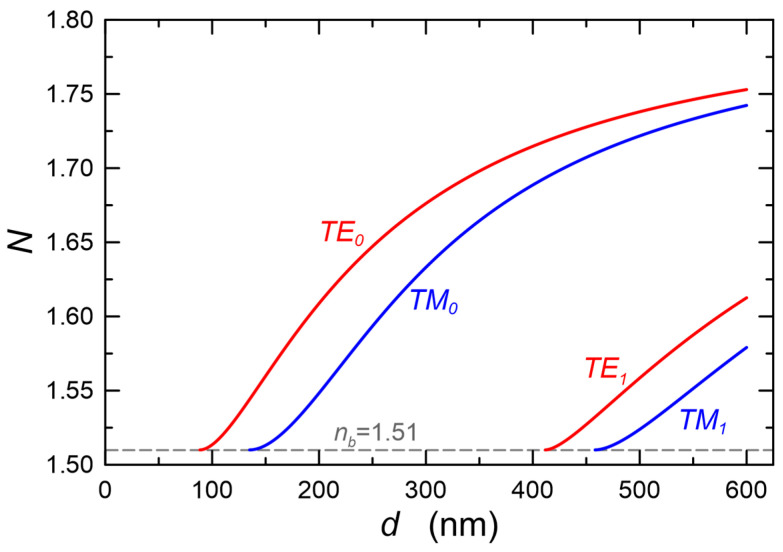
Modal characteristics of slab waveguides. *n_b_*/*n*_1_/*n_c_* = 1.51/1.80/1.00, *λ* = 632.8 nm.

The calculations presented here were carried out using the Berreman 4 × 4 matrix [59]. Figure 2 shows the calculated mode characteristics of the three-layer optical waveguide structure with the refractive indexes *n_b_*/*n*_1_/*n_c_* = 1.51/1.80/1.00, which correspond to the structures produced by us, presented in the experimental part of this article.

**Figure 3:**
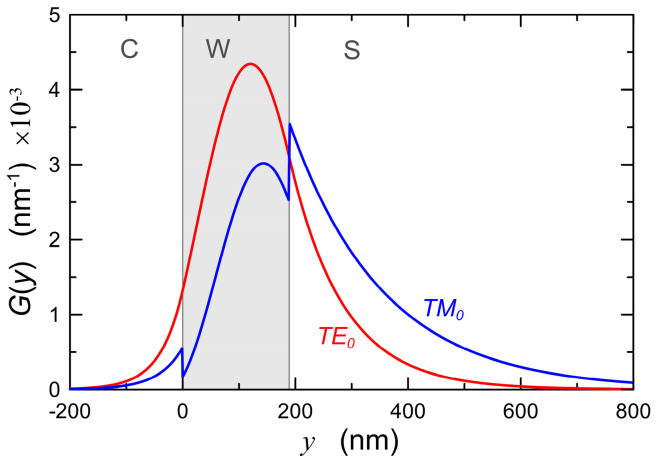
Distributions of the normalized power density in the slab waveguide. *n_b_*/*n*_1_/*n_c_* = 1.51/1.80/1.00, d = 190 nm, *λ* = 632.8 nm.

The calculations were made for the wavelength *λ* = 632.8 nm. In the thickness range *d* = 135–412 nm, the optical waveguide is single-mode and supports both basic modes TE_0_ and TM_0_. Exemplary distributions of normalized optical power density *G*(*y*) for a waveguide layer with a thickness *d* = 190 nm are presented in Figure 3. These distributions are normalized to the unit power guided by the mode per unit length in the direction *z* (Figure 1). The distributions of power density in planar evanescent wave sensors determine the part of the power of the guided mode which interacts with the cover or with the sensor layer, and thus they have impact on the optical sensitivity as well as on scattering losses at the interfaces (Equation (9)) and on the scattering losses on nanocrystals/pores in the volume of the waveguide layer (Equation (12)).

**Figure 4:**
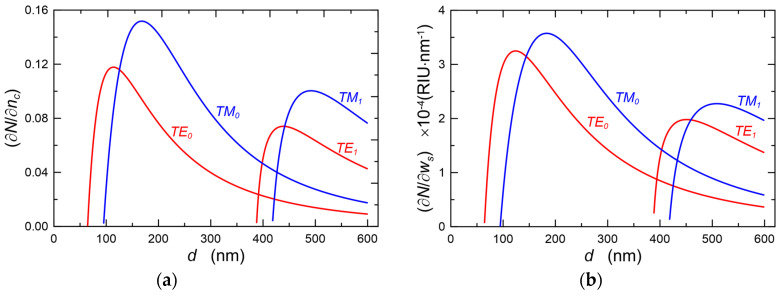
Optical sensitivities of waveguide structure with refractive indexes *n_b_*/*n*_1_/*n_c_* = 1.51/1.80/1.33, *λ* = 632.8 nm. (**a**) homogeneous sensitivity, (**b**) surface sensitivity, *w_s_* = 1 nm, *n_ws_* = 1.50.

Exemplary dependences, respectively of homogeneous sensitivity $\left(\partial N/\partial n_{c}\right)$ and surface sensitivity $\left(\partial N/\partial w_{s}\right)$ on the thickness of the waveguide layer are presented in Figure 4. Characteristics are calculated for the refractive indexes *n_b_*/*n*_1_/*n_c_* = 1.51/1.80/1.33 and for the wavelength *λ* = 632.8 nm, for the fundamental modes TE_0_, TM_0_ and for the first-order modes TE_1_, TM_1_. In the second case (Figure 4b), it was assumed that the sensor layer has a thickness *w_s_* = 1.0 nm and its refractive index *n_ws_* = 1.50, while the refractive index of the cover is the same as in the first case *n_c_* = 1.33. As we can see, for each of the considered modes, optical sensitivities assume maximum values for the thickness of the waveguide layer slightly greater than the cut-off thickness. As we can observe, the maxima of surface sensitivities occur at the thicknesses *d* of the waveguide layer slightly greater than the maxima of the homogeneous sensitivities $\left(\partial N/\partial n_{c}\right)$.

**Figure 5:**
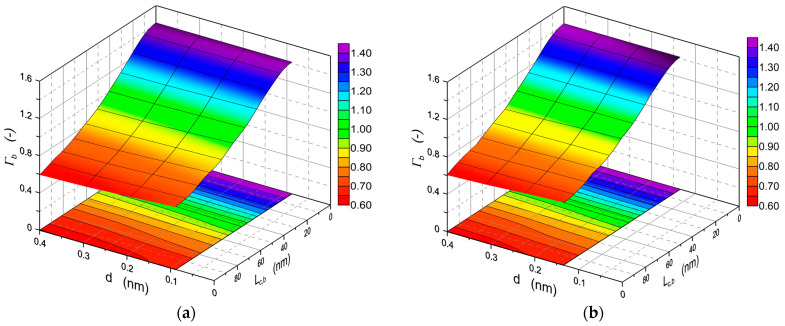
Influence of waveguide film thickness *d* and autocorrelation length *L_c,b_* on the factor *Γ*. (**a**) TE_0_ mode, (**b**) TM_0_ mode, *λ* = 632.8 nm.

The Lacey-Payne model has been derived for the TE polarization modes, and it is commonly used for such cases, mainly to calculate the losses in strip waveguides resulting from the sidewall roughness [72,73,74,75]. As we can see, the expression (9) for the attenuation coefficient is the product of power density on the surface of the waveguide layer ($\Phi_{\xi }^{2}$), wavenumber in free space (*k*_0_), parameters characterizing the waveguide optics structure (*Δ_ξ_*, *n*_1_) and the surface of the waveguide layer (*σ_ξ_*, *L_c,__ξ_*) as well as the dimensionless factor *Γ_ξ_*. It is easy to notice that for a given waveguide layer, for the steady-state wavelength *λ* and polarization state, the dimensionless factor *Γ_ξ_* depends only on the thickness of the waveguide layer and on the length of the autocorrelation path *L_ξ_*. The calculated dependences of the dimensionless factor *Γ_b_* on the thickness of the waveguide layer *d* and on the autocorrelation length *L_c,b_* for both basic modes TE_0_, TM_0_ are presented in Figure 5. In both cases, the same maximum value *Γ_b,max_* = $\sqrt{2}$ is obtained for zero autocorrelation length (*L_c,b_* = 0), and it remains constant for all values of *d*. For non-zero autocorrelation lengths *L_c,b_* there is a slight dependence of the dimensionless factor *Γ_b_* on the thickness of the waveguide layer—*Γ_b_* is decreasing with the rise of *d*. Ultimately, for *L_c,b_* = 100 nm, for the TE_0_ mode, the *Γ_b_* factors decrease from the value of 0.670 for the cut-off thickness of this mode to the value of 0.594 for the thickness *d* = 400 nm. In addition, for the TM_0_ mode, the value *Γ_b_* decreases from 0.670 to 0.604. Thus, it can be seen from the above that the influence of the thickness *d* of the waveguide layer on the dimensionless factor *Γ_b_* is negligible. This means that in the Formula (9) for the attenuation factor, practically only the optical power density $\Phi_{\xi }^{2}$ depends on light polarization. We accept, therefore, that this formula can also be used for the TM polarization. The validity of this assumption will be confirmed in Section 5.5. Due to the leap of power density of TM modes at the interface of the waveguide layer (Figure 3), we adopt for the calculations the average value of power density on both sides of the interface.

**Figure 6:**
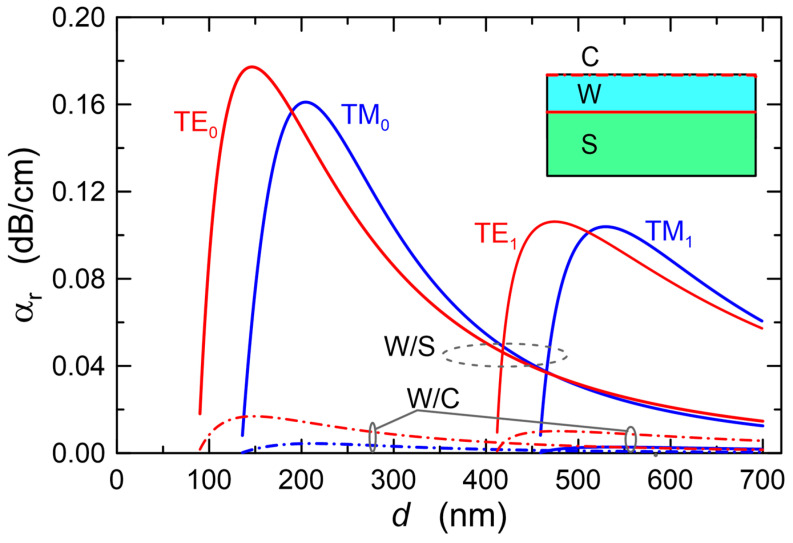
Optical scattering loss for first two modes versus waveguide film thickness. *L_c,b_* = 72 nm, *σ_b_* = 0.5 nm, *L_c,c_* = 10 nm, *σ_c_* = 0.2 nm, *n_b_*/*n*_1_/*n_c_* = 1.51/1.80/1.00, *λ* = 632.8 nm.

The dependences of scattering losses on the thickness *d* of the waveguide layer for the modes TE_0_ and TE_1_ as well as TM_0_ and TM_1_ calculated with the use of extended Lacey-Payne model are plotted in Figure 6. The calculations were made for the wavelength *λ* = 632.8 nm and for the refractive indexes *n_b_/n*_1_*/n_c_* = 1.51/1.80/1.00. The values of the parameters describing the surface morphology, respectively of the waveguide layer (W/C) and substrate (W/S) which were adopted for calculations are similar to those determined for real structures, presented in Section 5. Continuous lines are used to plot scattering losses, whereof source is the interface waveguide layer/substrate (W/S). In addition, dash-dot lines were used to plot scattering losses, whereof source is the interface waveguide layer/cover (W/C). Here, the scattering losses at the interface W/S for both polarizations are an order of magnitude greater than the scattering losses at the interface W/C. It is effected by much higher optical power density at the interface W/S than that at the interface W/C (Figure 3), as well as by greater roughness (*σ_b_* > *σ_c_*) and length of the autocorrelation path (*L_c,b_* > *L_c,c_*) on the substrate surface than on the surface of the waveguide layer. Similarly, as in the case of optical sensitivities (Figure 4), the scattering losses for each mode have the maximum value at the thickness of the waveguide layer slightly above the cut-off thickness. With further rise in the thickness of the waveguide layer, the scattering losses at the interfaces of the waveguide layer decrease with the rise of its thickness. For the fundamental modes in the range of the waveguide layer thickness above the cut-off of first-order modes (*d* > 450 nm), the calculated scattering losses reach the level even an order of magnitude lower than the maximum values. The nature of the dependence of the scattering losses at the interfaces of the waveguide layer on its thickness is similar to the dependence of optical sensitivities on the thickness of the waveguide layer, presented in Figure 4. In each case, both scattering losses and optical sensitivities reach their maximum values for the thickness of the waveguide layer slightly greater than the cut-off thickness. This means that the sensor structure with the optimal thickness from the viewpoint of optical sensitivity will be characterized by maximum scattering losses at the interfaces of the waveguide layer. Transition metal oxides (TiO_2_, ZnO, HfO_2_, ZrO_2_) have high refractive indexes, and hence they offer the potential possibility of attaining high optical sensitivities. However, due to the tendency to crystallize, the layers produced from them are polycrystalline with relatively high surface roughness. As a result, scattering losses in such layers, for thicknesses that are optimal from the viewpoint of optical sensitivity, can reach the level of even several dozen dB⋅cm^−1^. It can be seen from the computational characteristics shown in Figure 5 that even for a waveguide layer with a relatively high roughness, low scattering losses *α_r_* can be achieved for the fundamental mode when this optical waveguide is already a multimode one. In thicker waveguide layers, the field of the fundamental mode is concentrated inside the layer, and power densities on its boundary surfaces are then relatively low, but then, as shown below, scattering losses resulting from the scattering on nanocrystals contained in the waveguide layer may become significant.

**Figure 7:**
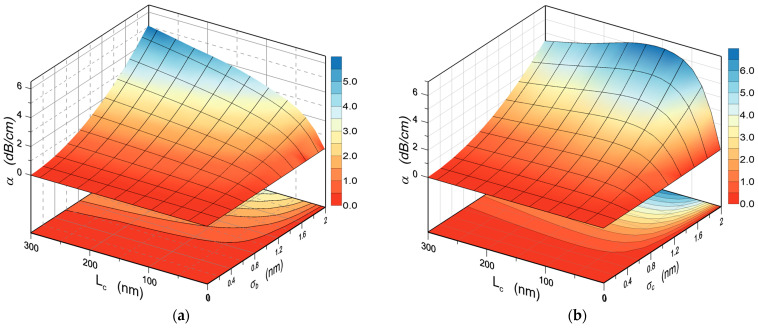
Influence of autocorrelation length and surface roughness on scattering losses of TE_0_ mode for refractive indexes *n_b_*/*n*_1_/*n_c_* = 1.51/1.80/1.00 and wavelength *λ* = 632.8 nm. (**a**) interface waveguide layer/substrate (W/S), (**b**) interface waveguide layer/cover (W/C).

The relationships binding the scattering losses *α_r_* with the autocorrelation length *L_c_* and roughness *σ_b,c_*, for the interface W/S (a) and W/C (b) are presented in Figure 7. Characteristics are calculated for the TE_0_ mode. In the first case (Figure 7a), the scattering losses increase monotonically both with the rise of the autocorrelation length *L_c_* and roughness *σ_b_*. In the second case, the scattering losses are a non-monotonic function of the autocorrelation length *L_c_*.

**Figure 8:**
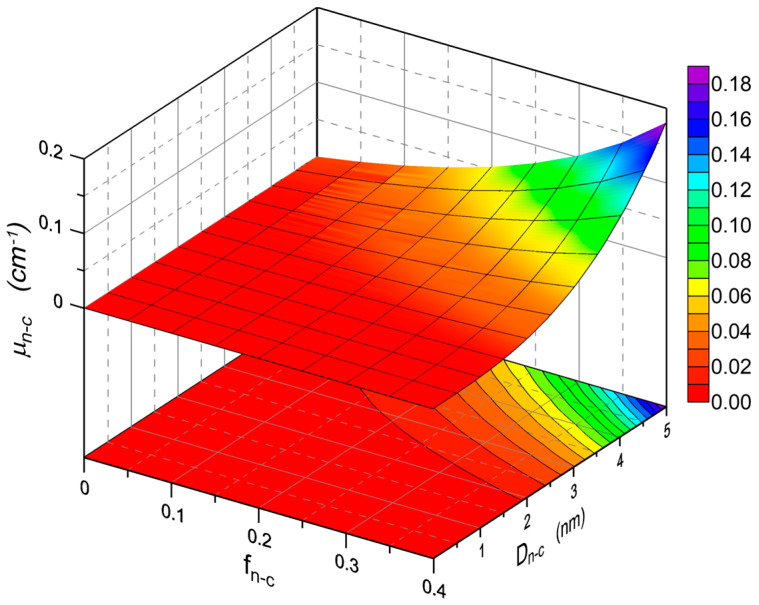
Influence of volume fraction of the anatase nanocrystals and their diameter on exponential radiation loss coefficient. *λ* = 632.8 nm.

When the material of the waveguide layer is polycrystalline, scattering of light on the nanocrystals should also be allowed for in the loss analysis. The impact of the content of anatase nanocrystals (*n*_2_ = 2.52) in the SiO_x_:TiO_y_ composite material and that of their diameter *D_n−c_* on the exponential radiation loss coefficient is illustrated by the calculation results presented in Figure 8. The calculations assume that the refractive index of the SiO_x_:TiO_y_ composite material is constant, it is independent of the content of nanocrystals and equals *n* = 1.8. Thus, along with the change of the volume fraction of crystalline particles, the refractive index of the SiO_x_:TiO_y_ matrix, in which the anatase nanocrystals are suspended, is changing. The refractive index of this matrix was calculated from the Lorenz-Lorentz relationship. As it can be observed, as expected, the exponential radiation loss coefficient increases both with the rise of the size of the nanocrystals *D_n−c_* and with their volume fraction *f_n−c_*.

**Figure 9:**
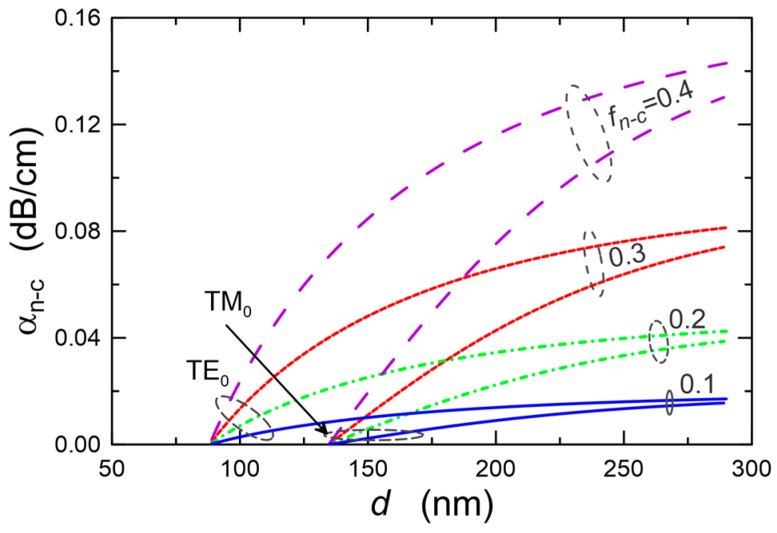
Influence of waveguide film thickness on scattering loss for selected volume fraction of the anatase nanocrystals of diameter equal 3 nm. *λ* = 632.8 nm.

The maximum value of the loss coefficient here is 0.18 cm^−1^. The value of the volume fraction *f_n−c_* corresponds to a situation where almost all of the titanium oxide contained in the SiO_x_:TiO_y_ material (Si:Ti = 1:1) would be in the form of anatase nanocrystals with a diameter *D_n−c_* = 5 nm. Figure 9 shows the dependences of optical losses *α_n−c_* on the thickness *d* of the waveguide layer for the selected values of volume fraction *f_n−c_* of crystalline particles and on the diameter of the nanoparticles *D_n−c_* = 3 nm, calculated for the basic modes TE_0_ and TM_0_. This value of *D_n−c_* was selected for the calculations because it was obtained from the experimental data using the Tauc method, which is presented in Section 5.2. As it can be seen, for both fundamental modes, the calculated optical losses increase monotonically with the increase of the thickness *d* of the waveguide layer. It is affected by the monotonic rise of the part of the optical power propagating in the waveguide layer when its thickness increases. The waveforms of the optical sensitivity characteristics presented in Figure 4 demonstrate that from the viewpoint of sensor applications, the optimal thicknesses of the waveguide layers analyzed here have the value ca. 200 nm. The calculation results presented in Figure 9 show that if all the titanium oxide contained in the composite waveguide layer SiO_x_:TiO_y_ (Si:Ti = 1:1) was in the form of anatase nanocrystals with a diameter *D_n−c_* = 3 nm, then the scattering losses for the TE_0_ mode would be below 0.12 dB⋅cm^−1^. This level of optical loss resulting from light scattering on nanocrystals can be considered acceptable. Yet surely, such a situation (*f_n−c_* = 0.4) would also result in a relatively high roughness of the surface of the waveguide layer *σ_c_*. As a result, the level of optical losses resulting from light scattering on the surface of the waveguide layer could be significantly higher than the level of scattering losses on nanocrystals. From the results presented in Figure 8 and Figure 9, one can observe that the optical scattering losses significantly decrease with the decreasing volume fraction *f_n−c_* of nanocrystalline particles.

## 3. Waveguide Film Fabrication

The processes of sol-gel technology are described in detail in a comprehensive book; “Sol-Gel Science” by Brinker and Scherer [46]. Two-component composite waveguide layers SiO_x_:TiO_y_ were produced from a sol whereof synthesis was made with tetraethyl orthosilicate Si (OC_2_H_5_)_4_ (TEOS) and tetraethyl orthotitanate Ti (OC_2_H_5_)_4_ (TET) as the precursors, respectively of silica and titania. The other ingredients included deionized water, ethyl alcohol acting as a homogenizing agent and hydrochloric acid, which was used to catalyze the reactions of hydrolysis and condensation. The procedure for the fabrication of the two-component sol was a two-step process. In the first stage the hydrolysis of TEOS and TET was carried out separately in ultrasonic fields (~40 kHz, 120 W). Then partially hydrolyzed TET solution was added to partially hydrolyzed TEOS solution, and the sol formation process was carried out. This process was also carried out in the ultrasonic field. The solutions were mixed in proportions ensuring that the molar ratio Si:Ti = 1:1. The resulting solution was homogeneous, transparent, and light yellow in color. Waveguide films were coated on soda-lime microscope slides (Menzel Glaser) and BK7 glass substrates (76 × 26 × 1 mm^3^). For measurements with SEM/TEM methods, layers were also produced on silicon substrates. The substrate plates were washed following the procedure which included: mechanical cleaning in water with detergent, rinsing in deionized water, rinsing in acetone and drying.

In our studies the dip-coating method was applied, in which substrate withdrawal speed *v* from the sol is the basic parameter having the influence on the thickness of the obtained film. When the sol shows the properties of Newtonian liquid and its viscosity and substrate withdrawal speed *v* are high enough to lower the curvature of meniscus, then the dependence of the thickness *d* of the sol film is proportional to *v*^1/2^ [46,47,76,77]. Whereas then the movement of substrate does not result in the change of meniscus curvature, the thickness *d* is proportional to *v*^2/3^ [46,47,76,77]. Finally, the fabricated structures were heated for 1 h at temperature 500 °C.

## 4. Measurements Methods and Instrumentation

### 4.1. Thickness and Refractive Index

The thickness and refractive index of the sol-gel derived silica-titania waveguide films have been determined by ellipsometry way. Ellipsometry technique uses light of known polarization incident on a structure under study and detects the polarization state of the reflected light [78]. Incident light is usually linearly polarized and reflected light has elliptical polarization. The fundamental equation of ellipsometry has the following form:

(13) $$\rho =\frac{r_{p}}{r_{s}}=\tan\left(\psi \right)\exp\left(i\Delta \right)$$

where *r_p_*, *r_s_* are the Fresnel complex amplitude reflection coefficients for light polarized parallel, perpendicular to the plane of incidence, respectively. The angles *ψ* and Δ are the standard ellipsometry angles representing the change in amplitude and in phase shift, respectively, upon reflection. The fundamental ellipsometry Equation (13) allows one to determine the thickness *d* of a film, the refractive index *n* and extinction coefficient *κ*. At the stage of technological research, the SE400 monochrome ellipsometer (Sentech, Germany) was used to determine the thickness *d* and the refractive index *n* of the produced layers. For spectral characterization of the produced layers, the spectroscopic ellipsometer Woollam M-2000 (J.A.Woollam) and the program CompleteEASE were used. In ellipsometry spectroscopic measurements, an extremely important issue which has impact on the quality of the obtained results is the selection of an appropriate dispersion model of dielectric functions. Having tested various models, we selected the Tauc-Lorentz model as the most appropriate one [79]. This model well describes such materials as semiconductors [80], insulators [81], and polymers [82]. Tauc-Lorentz model calculates the imaginary part of the dielectric function *ε^(i)^* by multiplying the Tauc joint density of states [83] and the $\varepsilon_{L}^{\left(i\right)}$ obtained from the Lorentz oscillator model. We used the two-oscillator Lorentz model, hence the expression for the imaginary part of the dielectric function *ε^(i)^* has the form:

(14) $$\varepsilon^{\left(i\right)}=\{\begin{array}{cc} \sum_{j=1}^{2}\frac{A_{j}\cdot E_{j}\cdot C_{j}\cdot {\left(E-E_{g}\right)}^{2}}{{\left(E^{2}-E_{j}^{2}\right)}^{2}+C_{j}^{2}\cdot E^{2}}\times \frac{1}{E} & \begin{array}{ccc} & for & E>E_{g} \end{array}\\ 0 & \begin{array}{ccc} & for & E\leq E_{g} \end{array} \end{array}$$

The seven fitting parameters are *E_g_*, *A*_1,2_, *E*_1*,*2_, and *C*_1*,*2_, and all are in units of energy. The real part of the dielectric function *ε^(r)^* is derived by Kramers-Kronig integration:

(15) $$\varepsilon^{\left(r\right)}\left(E\right)=\varepsilon^{\left(r\right)}\left(\infty \right)+\sum_{j=1}^{2}\frac{2}{\pi }\cdot P_{c}\cdot \int_{E_{g}}^{\infty }\frac{\xi \cdot \varepsilon_{j}^{\left(i\right)}(\xi )}{\xi^{2}-E^{2}}d\xi$$

where the *P_c_* stands for the Cauchy principal part of the integral and eight fitting parameter $\varepsilon^{\left(r\right)}\left(\infty \right)$ is the high frequency dielectric constant. The solution of this integral in the form of an analytical function is given in Ref. [79].

### 4.2. Transmission Properties, Energy Gap and Optical Homogeneity

The transmittance and reflectance characteristics of the produced SiO_x_:TiO_y_ composite layers were recorded using a UV-Vis AvaSpec-ULS2048LTEC Spectrophotometer (Avantes) and an AvaLight-DH-S-BAL (Avantes) light source. Optical spectra were recorded in the wavelength range of 200–1100 nm. Based on the reflectance spectra, the optical homogeneity of SiO_x_:TiO_y_ composite layers was assessed using the method described in our earlier work [63]. In the spectral range away from the absorption edge, the reflectance spectra recorded for the substrate and for the substrate with the applied SiO_x_:TiO_y_ layer are compared. When the minima of the reflectance characteristics of the SiO_x_:TiO_y_ layer lie on the reflectance characteristics of the substrate, this means that the layer is optically homogeneous. The optical band gaps *E_g_* were determined from the analysis of the transmittance spectra in the range of the absorption edge. In this spectral range, the energy of photons *hν*, the absorption coefficient *α* and the width of the optical band gap *E_g_* are combined with the Tauc relation [84]:

(16) $$\alpha \cdot h\nu =B{\left(h\nu -E_{g}\right)}^{\chi }$$

where *B* is a constant which does not depend on photon energy *hν*, and χ is the power coefficient which value determines the type of optical transition. The power coefficient χ takes the value 2 for an indirect allowed transition and the value 0.5 for a direct allowed transition. The linear dependence of (*α*⋅*hν*)^1/^^χ^ on photon energy and its extrapolation to (*α*⋅*hν*)^1/^^χ^ = 0, give the values of the optical band gap for indirect allowed transition $E_{g}^{ind}$ (*χ* = 2) or for direct allowed transition $E_{g}^{dir}$ (*χ* = 1/2), respectively.

### 4.3. Morphology of the SiO_x_:TiO_y_ Composite Films

Surface morphology of the obtained composite SiO_x_:TiO_y_ films was studied using AFM method. The AFM studies were carried out using PSIA XE-70 (Park System Corp., Suwon, Korea) working in non-contact mode. The BS Tap300Al cantilevers (resonance frequency 300 kHz, force constant 40 N/m) were used. Acquired images were processed using image processing software (XEI^®^, PSIA and Gwyddion^®^ software) to correct sample inclination and distortions caused by z-scanning stage. No other corrections in images were made.

#### 4.3.1. SEM/FIB

SEM images and FIB cross-section were obtained using Helios NanoLab 660 microscope equipped with Schottky electron source, Ga^+^ ion source and gas injection system (GIS) for platinum deposition. Prior to the microscopic examination of a sample on glass substrate, the thin carbon layer (about 15 nm) was evaporated on the sample in order to reduce the charging effect under electron beam. Additionally, prior to the cross-sectioning, the platinum layer was locally deposited as a protective layer against damage caused by ion beam. The FIB cross-section was made by ion beam milling.

#### 4.3.2. TEM Specimen Preparation

Prior to FIB-lamella preparation the sample surface was sputtered with ~60 nm thick amorphous carbon film to serve as protection layer. Cross-sectional FIB-lamella is prepared by standard Focused Ion-Beam (FIB) milling technique on ThermoFisher Scientific Helios NanoLab 450HP SEM/FIB microscope equipped with Ga^+^ ion gun. The final thinning step of the lamella was carried out at 1 kV, 29 pA beam current.

#### 4.3.3. S/TEM Characterization

Scanning and Transmission electron microscopy (S/TEM) characterization was performed in ThermoFisher Scientific Titan 60–300 cubed microscope equipped with high brightness X-FEG gun, monochromator, double Cs corrector (image Cs-corrector, DCOR probe Cs-corrector), ChemiSTEM super-X EDS 4-detectors system and Gatan continuum EELS spectrometer. The sample was investigated using the 300 kV accelerating voltage. STEM-HAADF imaging was performed with beam current of 80 pA and beam convergence angle was 21.4 mrad, and HAADF detector collection angle 50.5–200 mrad.

### 4.4. Waveguide Properties

**Figure 10:**
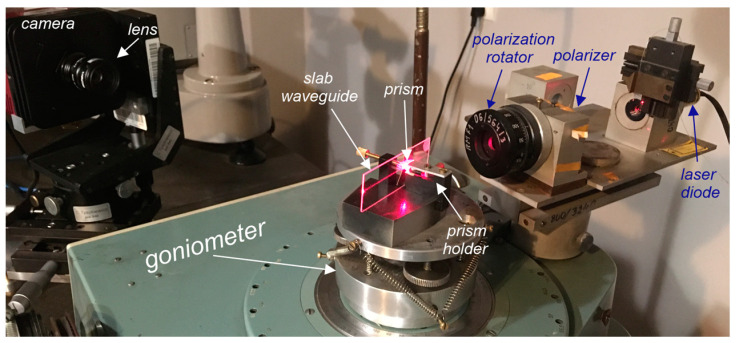
Set-up for optical loss measurement.

The waveguide properties were investigated using the *m*-line method in the goniometric system presented in Figure 10. The optical waveguides were excited with the use of a prism coupler using the optical tunneling effect. The excitation characteristics of the slab waveguides and trails of scattered light were recorded, on the basis of which optical losses in the composite waveguide layers SiO_x_:TiO_y_ were determined. Light propagation in the optical waveguide is accompanied by its scattering, whereof potential sources were discussed in Section 2 (Figure 1). Consequently, a trail of scattered light is visible in the excited optical waveguide. By recording its image and by analyzing the intensity distribution of the scattered light along the direction of its propagation, it is possible to determine propagation losses. The streak method assumes that the intensity of the scattered light at each point in the streak is proportional to the intensity of the light propagating at that point in the waveguide layer. Moreover, homogeneity of the waveguide layer in the direction of light propagation is assumed. Therefore, we can state that the distribution of the intensity of the scattered light in the streak changes in line with the relation *I*(*x*) = *I_0_*⋅exp(−*μx*). By approximating light intensity distribution in the registered streak with the use of this dependence, the attenuation coefficient *μ* is determined, and then the propagation losses *α* = 4.34⋅*μ* (dB⋅cm^−1^) are calculated. This procedure is carried out with selective excitation of individual modes, which can be easily carried out using a goniometer, a polarizer and a polarization rotator. The streak method also assumes the homogeneity of the substrate on which the waveguide layer is applied. This assumption is not always met, especially when the substrate surface has been prepared by grinding and polishing processes. Any point defects of the substrate or scratches which result in strong point scattering of light (Mie scattering) adversely affect the measurement result with the use of the streak method. The basic requirement underlying the measurement method discussed here is the necessity to register a streak of scattered light, which in some cases may be difficult. The image of the excited optical waveguide is registered from the direction of the normal to its surface. In the case of very good quality of optical waveguides, it may be difficult to register such a streak, despite the introduction of significant optical power to the optical waveguide. Due to non-isotropic nature of the scattering of light guided in the optical waveguide, with a very good optical waveguide, we can visually observe at large angle a clear streak of scattered light, while when it is recorded with a camera from the direction of the normal, it will have low light intensity. Hence, the limitations of the streak method as well as difficulties in its application.

The measurement system for recording streaks of scattered light emitted by excited planar waveguides is shown in Figure 10. The examined structures were placed on the goniometer stage, thanks to which it was possible to set the correct angle of illumination. A laser diode operating at the wavelength *λ* = 676.7 nm was used as a light source. The modes guided in the planar waveguide were excited with the use of a prism coupler and in some cases with the use of grating couplers. The use of the prism coupler does not require any preparation of the slab waveguide for measurements. The correct polarity was set using a polarizer and a polarization rotator. The streak of scattered light was recorded with a CCD camera (1501M-USB-TE—1.4 Megapixel Monochrome Scientific CCD Camera, Thorlabs) with an appropriately selected lens, which ensured the acquisition of an image of the tested waveguide on a camera chip of the size similar to the size of the chip. The light streaks were recorded on a computer, which is not shown in Figure 10. In the same goniometric system, the excitation characteristics of waveguide modes (*m*-line spectra) were recorded, i.e., the dependences of the signal at the output of the planar waveguide on the illumination angle of the prism or grating coupler. The effective refractive indexes of the excited modes were calculated from the angular position of the coupling peaks.

## 5. Experimental Results and Discussion

### 5.1. Film Thickness and Refractive Index versus Substrate Withdrawal Speed

**Figure 11:**
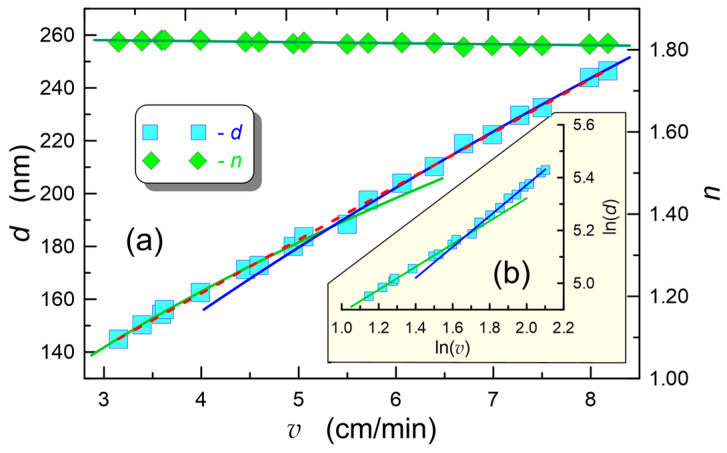
Influence of substrate withdrawal speed from sol on final film thickness and refractive index (a), and film thickness vs substrate withdrawal speed, in logarithmic scale (b).

Exemplary characteristics involving the influence of substrate withdrawal speed on the final values of the thickness and refractive index of the composite layers SiO_x_:TiO_y_ are presented in Figure 11a. The values of thickness and refractive index on this figure are marked with squares and diamonds, respectively. The experimental characteristic of the refractive index depending on substrate withdrawal speed *v* was approximated by a linear function, which yielded *n*(*v*) = (1.8301 ± 0.0008) − (0.0025 ± 0.0002 cm^−1^s) *v*. It can be seen from the above that the refractive index slightly decreases with the rise of substrate withdrawal speed *v*. The dependence of the final layer thicknesses *d* on the substrate withdrawal speed *v* was also approximated by a linear function and plotted in Figure 11b with a dashed red line. As one can see, a good fit of the linear function to the experimental points was obtained. Thus, we can accept that in this wide range of speeds *v*, the relation *d*(*v*) is linear. However, when plotting *d*(*v*) on a log scale, as shown in Figure 11b, one can see two ranges of speeds with different slopes of the experimental characteristics *d*(*v*). These ranges in the discussed case are parted by the speed *v* ~5.5 cm/min. From the approximation of the experimental points with linear functions in these ranges, the values of slopes *γ* = 0.484 ± 0.002 (green line) and *γ* = 0.651 ± 0.002 (blue line) were obtained.

In Figure 11a, the corresponding curves for these slopes are plotted with lines of the same color as in Figure 11b. One can observe that in the speed range *v* < 5.5 cm⋅min^−1^, the movement of the substrate does not result in the change of the curvature radius of the meniscus at the sol-substrate interface. In addition, above this speed, the movement of the substrate reduces the radius of curvature of the meniscus at the sol-substrate interface. As one can see, in this case slope values *α* are almost identical to those obtained from the models presented in Section 3.

The average value of the refractive index calculated for the SiO_x_:TiO_y_ waveguide layers presented in Figure 11 is *n*_1_ = 1.817. The measured refractive index of the layer made of the same sol on silica substrate and annealed at the temperature of 800 °C for 60 min was 1.90. In addition, the refractive index of the compact layer SiO_x_:TiO_y_ with a molar ratio of Si:Ti = 1:1 calculated from the Lorenz-Lorentz formula is *n_d_* = 1.914. This refractive index was allowed for when calculating the residual porosity *P* for the layers annealed at 500 °C, using the Lorenz-Lorentz formula:

(17) $$\frac{n_{1}^{2}-1}{n_{1}^{2}+2}=(1-\frac{P}{100\%})\frac{n_{d}^{2}-1}{n_{d}^{2}+2}$$

Hence, the calculated residual porosity *P* = 7.7%.

### 5.2. Optical Band Gap and Homogeneity

**Figure 12:**
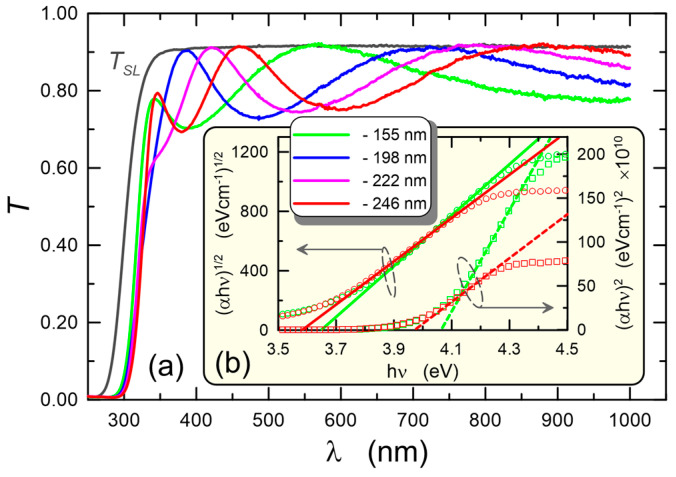
Transmittance characteristics for selected composite SiO_x_:TiO_y_ films and for soda-lime glass substrate (a), normalized absorption coefficients versus photon energy (b).

Spectrophotometric measurements can provide a lot of valuable information about the properties of the tested layers. Here we present the test results for the layers in Figure 12. The transmittance characteristics for structures with layers of different thicknesses are shown in Figure 12a. Gray color was used to plot the transmittance characteristic of soda-lime microscope glass slide. For each of the layers, the transmittance drops to zero for wavelengths below 300 nm, which is due to the presence of an absorption edge in this spectral range. The transmittance characteristics of the layers lie below the transmittance characteristics of the substrate, since the refractive indexes of the layers are higher than the refractive index of the substrate glass. Clear interference maxima and minima are visible. The interference maxima lie on the transmittance characteristics of the substrate, which indicates that the layers are homogeneous. Using the Tauc method, the optical band gaps were determined for indirect and direct transitions, respectively. Figure 12b presents the normalized absorptions ${\left(\alpha \cdot h\nu \right)}^{1/2}$ and ${\left(\alpha \cdot h\nu \right)}^{2}$ as a function of the energy of photons *hν* for layers with a thickness of 155 nm and 246 nm, respectively. For these layers, optical band gaps for indirect transitions were determined, which equal 3.582 eV and 3.648 eV, respectively, and which correspond to the wavelengths of 346 nm and 340 nm. Such high values of the optical band gaps indicate the amorphous nature of the produced SiO_x_:TiO_y_ composite layers. Assuming the presence of anatase nanocrystals in the SiO_x_:TiO_y_ composite layer, we find from the value of blue shift of the band-gap energy Δ*E* = 3.582 eV − 3.20 eV = 0.382 eV in quantum size effect, that the nanocrystalline diameters are not larger than *D_n−c_* = 3 nm [85].

**Figure 13:**
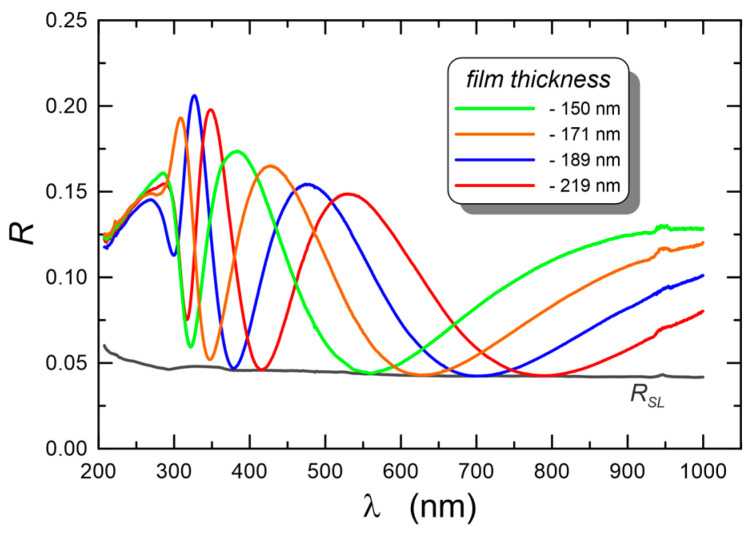
Reflectance characteristics for selected composite SiO_x_:TiO_y_ films and for soda-lime glass substrate.

The reflectance characteristics for selected structures with different layer thicknesses are presented in Figure 13. It can be seen that in the spectral range above the absorption edge, the interference minima of each of the characteristics lie on the reflection characteristics of the substrate. This fact evidently shows that the composite layers SiO_x_:TiO_y_ presented here have optical homogeneity [63].

### 5.3. Complex Refractive Index

**Figure 14:**
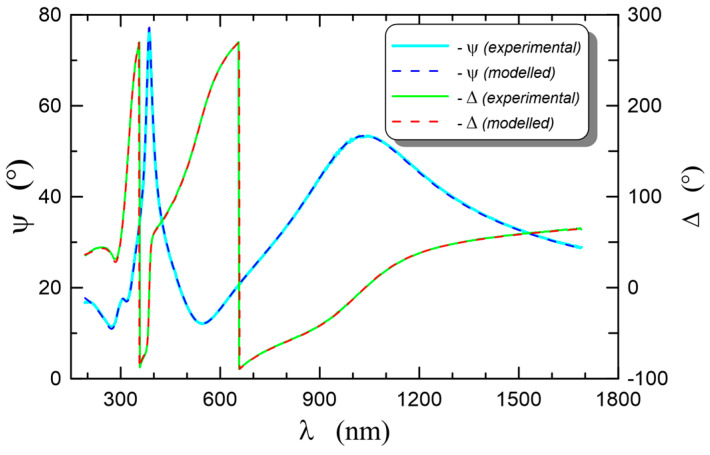
A typical spectrum of ellipsometry angles Ψ and Δ, measured for composite SiO_x_:TiO_y_ film on silicon substrate at 30 °C. The angle of illumination equals 70°.

In the procedure for determining spectral characteristics of the refractive and extinction indexes of the layer material, it is important to know the dispersion properties of the substrate. For this reason, a composite layer SiO_x_:TiO_y_ was prepared for ellipsometric spectroscopic studies on a silicon substrate, whereof optical properties are well known [86]. The measurement results of ellipsometry angles in the spectral range from 190 to 1700 nm, recorded at the illumination angle of the sample *θ* = 70°, are plotted with solid lines as presented in Figure 14. In addition, the dashed lines were used to plot dispersion characteristics calculated with the use of the Tauc-Lorentz formula presented in Section 4.1. These characteristics are the best fit for the experimental characteristics. In the applied Tauc-Lorentz model, two oscillators were taken into account, and hence, eight parameters were fitted. The calculated values of these parameters are summarized in Table 1. As shown in Figure 14, a perfect fit of the modeled dispersion characteristics of the ellipsometry angles to the experimental characteristics was obtained. Based on the calculations, the thickness of the SiO_x_:TiO_y_ composite layer on the silica substrate *d* = 171.7 nm was determined.

**Figure 15:**
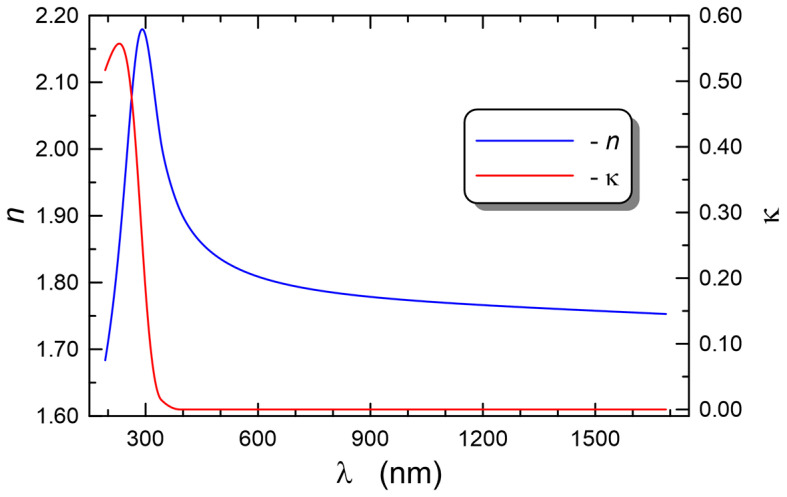
Dispersion characteristics of the refractive index *n* and extinction coefficient *κ* for composite SiO_x_:TiO_y_ film.

The dispersion characteristics of the refractive index *n*(*λ*) and those of the extinction coefficient *κ*(*λ*) corresponding to the best fitting of the modeled ellipsometry angle (Figure 14) are plotted in Figure 15. One can observe that the dispersion of the refractive index is normal in the spectral range above 300 nm, while below that wavelength, anomalous dispersion is observed, which is affected by the presence of the absorption band in this spectral range. For the wavelengths over 390 nm, the absorption disappears and the extinction coefficient *κ*(*λ*) = 0.

### 5.4. Film Morphology

As demonstrated in Section 2, from the viewpoint of the analysis of optical losses in planar optical waveguides, it is important to know both the morphology of the waveguide layer surface and the surface of the substrate on which it was applied. The main measurement method used for this purpose was atomic force microscopy, and the determined parameters included mainly surface root mean square roughness of the surface *σ* and autocorrelation length *L_c_*.

**Figure 16:**
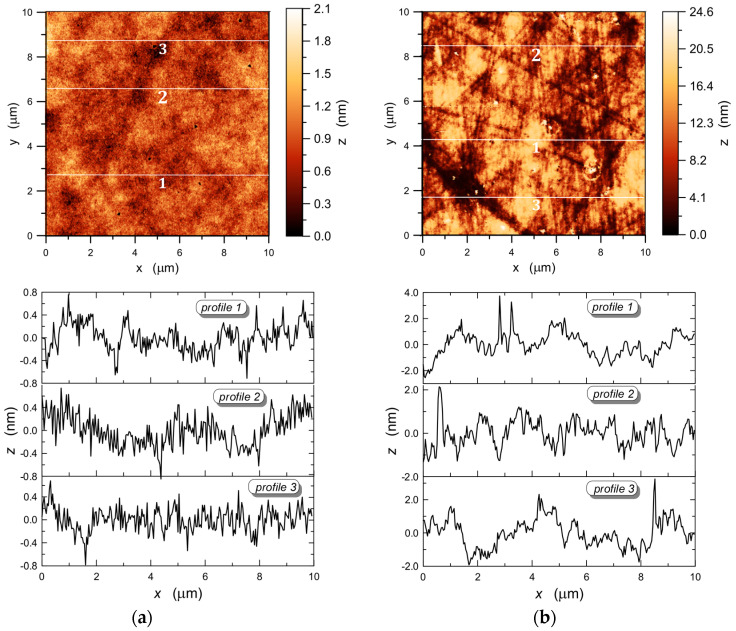
AFM images and corresponding profiles for composite SiO_x_:TiO_y_ film (**a**) and BK7 glass substrate (**b**).

Exemplary AFM 10 × 10 μm^2^ images, respectively of the surface of the SiO_x_:TiO_y_ composite layer and the BK7 substrate glass are shown in Figure 16. Scan lines along which the surface profiles were determined were marked on these images. The surface profiles corresponding to the individual scan lines are shown below the respective AFM images. We can see that the surface of the composite layer is much smoother than that of the BK7 substrate glass. This is obvious since the layer was made of a liquid phase, while the substrate glass was cut out from the BK7 glass block and then ground and polished. In the AFM image of the surface of BK7 substrate glass, scratches effected by its mechanical processing are visible. The differences in height between the highest and the lowest points are, respectively, 1.468, 1.586 and 1.461 nm for the surface profiles 1, 2 and 3 of the SiO_x_:TiO_y_ composite layer, and standard deviations calculated from these profiles are respectively *σ* = 0.24 nm, 0.28 nm and 0.20 nm. The difference in height between the highest and the lowest point on the presented surface 10 × 10 μm^2^ of the composite layer is 2.1 nm, and the standard deviation determined for this area is *σ* = 0.29 nm, and as we can see it is at the level of standard deviations calculated from individual profiles (Figure 16a). This bespeaks of high homogeneity of the SiO_x_:TiO_y_ composite layer.

In the case of the substrate glass BK7, whereof AFM image is shown in Figure 16b, for profiles 1, 2 and 3, the differences in height between the highest and the lowest points are respectively 6.48 nm, 3.46 nm and 5.17 nm, and the standard deviations calculated from these profiles are respectively *σ* = 0.96 nm, 0.62 nm and 0.88 nm. In the entire imaged area of 10 × 10 μm^2^ of the substrate glass BK7, the difference in height between the highest and the lowest point is over 24 nm, while the standard deviation calculated for the entire area is 1.03 nm. The correctness of the AFM method is validated by the reduction of the determined roughness *σ* along with the reduction of the scanned area, while maintaining a constant resolution.

**Figure 17:**
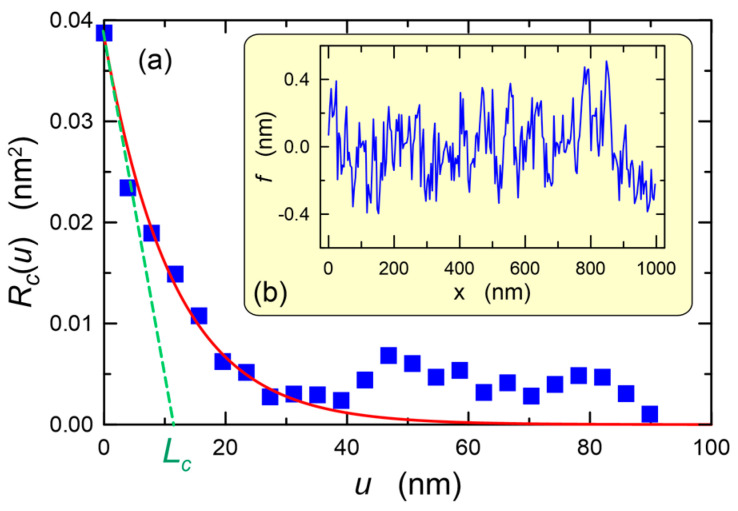
Autocorrelation function of top surface of the waveguide film. *L_c_* = 11.4 nm, *σ* = 0.20 nm (a). Surface profile function (b).

Figure 17a presents the autocorrelation function marked with squares, determined for the upper surface of the SiO_x_:TiO_y_ composite layer from the surface profile shown in Figure 17b. This relationship was approximated by the exponential function (7), from which the autocorrelation length *L_c_* = 11.4 nm was calculated. The surface roughness determined from the applied profile *f(x)* is *σ* = 0.20 nm. For the BK7 glass substrate, the autocorrelation length of *L_c_* = 72 nm was determined using the same procedure.

**Figure 18:**
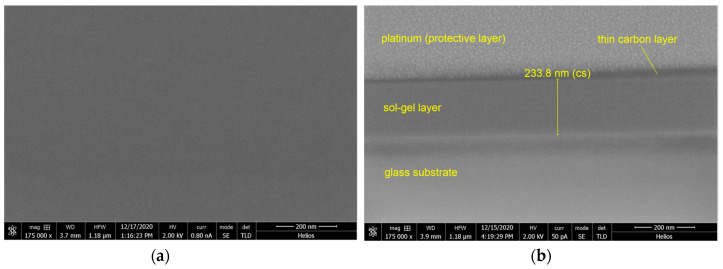
SEM images of top (**a**) and cross section (**b**) of the composite SiO_x_:TiO_y_ film.

The SEM image of the upper surface of the SiO_x_:TiO_y_ composite layer produced on glass substrate is shown in Figure 18a. We can see a smooth, crack free surface devoid of any defects. Figure 18b shows the SEM image of the cross-section of the structure. We can clearly see the 233.8 nm thick boundary surfaces of the layer, which are continuous and smooth. It can also be seen that the material of the layer is homogeneous throughout the entire cross-section.

**Figure 19:**
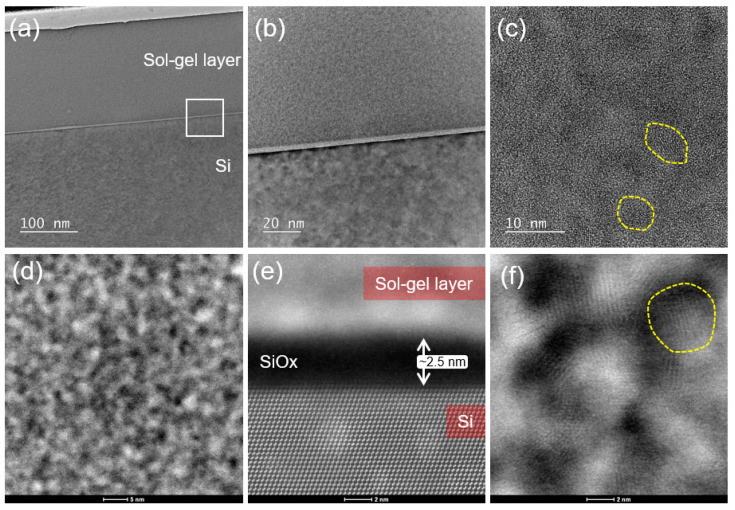
S/TEM characterization of the cross-section of composite SiO_x_:TiO_y_ layer on the silicon substrate. (**a**–**c**) TEM images; (**a**) overview TEM image of the sample cross-section, (**b**) magnified TEM image of the region corresponding to rectangle marker region in image (**a**), (**c**) HRTEM image of the sol-gel layer, representative regions of nano grains with lattice ordering are marked with yellow dotted line. (**d**–**f**) HR-STEM HAADF images; (**d**) shows the microstructure of sol gel layer, white speckle such as Z-contrast indicates homogenous distribution of Ti within the layer, (**e**) HR-STEM image of interface region between the sol-gel layer and Si substrate, the region with darker contrast corresponds to native oxide layer (SiO_x_), (**f**) HR-STEM image of the sol-gel layer showing the presence of ordered lattice in some nano-grains, one such representative region is marked with yellow dotted line.

**Figure 20:**
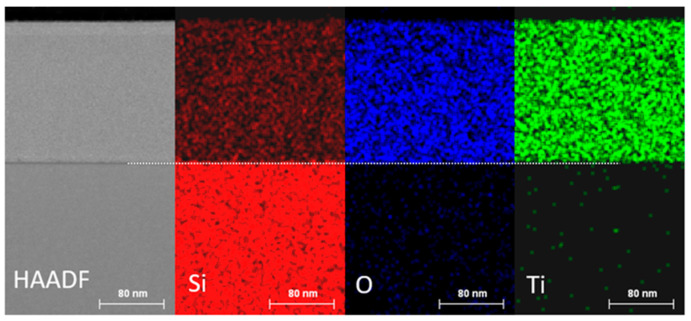
X-ray EDS mapping of the sample cross-section. The dark interface layer, is observed to be composed of only Si and O. This supports it is most likely the native oxide layer (SiO_x_) on Si substrate. The dotted line serves as a guide to an eye.

**Figure 21:**
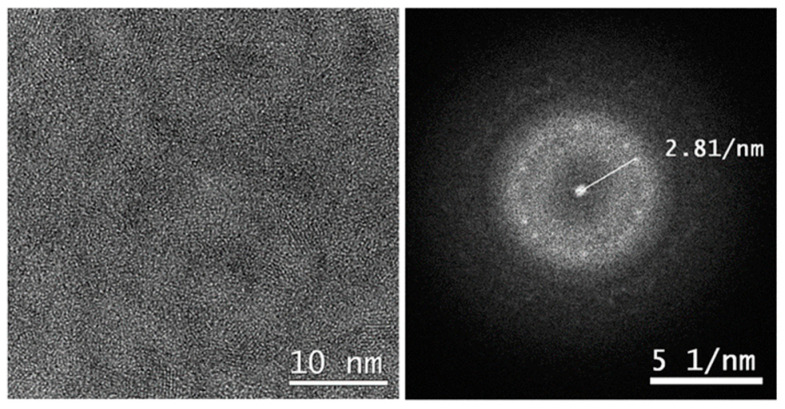
HRTEM image of the sol-gel layer and the corresponding FFT diffractogram. The FFT shows that sol-gel layer is predominantly amorphous with some randomly oriented crystalline nano grains with lattice d-spacing of 0.36 nm.

Microstructure of the composite SiO_x_:TiO_y_ layer on silicon substrate, in cross-section, was further investigated by HRTEM and HRSTEM characterization. The Figure 19a shows the TEM image of the sample cross-section, in this case the measured thickness of the sol-gel layer is 168 nm. The Figure 19b shows the magnified TEM image of the interface region marked by rectangular marker in image (a), the presence of an interface layer, the layer with bright contrast, between the sol-gel layer and the substrate is clearly visible. The HRSTEM-HAADF image of this region, as shown in Figure 19e, clearly shows the atomic lattice of Si substrate and that this interlayer has a darker contrast compared to Si substrate below and the sol-gel layer above, this is typical STEM-HAADF contrast of native oxide layer (SiO_x_) on Si. This is further supported by X-ray EDS elemental maps, see supplementary information Figure 20. The measured thickness of this layer was 2.5 nm. The Figure 19c shows the HRTEM image of the sol-gel layer, the image contrast is typical of amorphous microstructure and some crystalline regions (nano-grains) in the size range of 3.5–5 nm are observed, as shown by marked regions. This is further confirmed by the FFT diffractogram analysis of this image, see supplementary information Figure 21. The FFT diffractogram shows diffused halo, typical of amorphous microstructure, with a spotted ring. This ring in the FFT has a *d*-spacing of 0.36 nm and the spots support the presence of randomly oriented crystalline nano-grains. The HRSTEM-HAADF image of the sol-gel layer, Figure 19d, shows uniform distribution of brighter regions, based on the Z-contrast sensitivity of this imaging, this indicates homogenous distribution of the Ti-rich regions. The presence of the nano-grains with ordered lattice is additionally supported by HRSTEM-HAADF imaging, as shown by the marked regions in Figure 19f.

The EDS maps logged for the area marked in Figure 19a in the cross-section of the investigated structure are shown in Figure 20. We can observe even distribution of silica, oxygen and titania in the area of the SiO_x_:TiO_y_ layer.

### 5.5. Waveguide Properties

**Figure 22:**
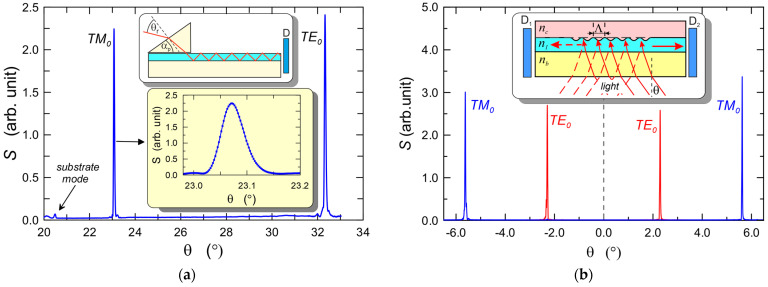
The *m*-line spectrum of the composite SiO_x_:TiO_y_ slab waveguide, recorded using prism coupler (**a**) and grating coupler (**b**). *λ* = 677 nm, D—photodetector.

Waveguide properties of the produced SiO_x_:TiO_y_ composite layers on glass substrates were tested in the goniometric system shown in Figure 10. An exemplary *m*-line spectrum recorded with the use of a prism coupler is shown in Figure 22a. The spectrum was recorded using a prism coupler made of SF-14 glass with a refractive index *n_p_* = 1.7099(3) and the breaking angle *α_p_* = 49.953(4)°. We can observe narrow peaks corresponding to the resonant excitation of the fundamental modes TM_0_ and TE_0_. These peaks occur at synchronous angles *θ_r_* equal to 23.0720° and 32.3273°, respectively. Hence, the calculated from the formula [87]:

(18) $$N=\sin\alpha_{p}\sqrt{n_{p}^{2}-\sin^{2}\theta_{r}}+\cos\alpha_{p}\sin\theta_{r}$$

effective refractive indexes of the basic modes have the values *N_TM0_* = 1.52626 and *N_TE0_* = 1.58737, respectively. Assuming that the layer is homogeneous, the refractive index of the layer *n*_1_ = 1.8095 and its thickness *d* = 182.5 nm were calculated from the system of characteristic Equation (1). The thickness is close to the optimum thickness in terms of optical sensitivity (Figure 4). Figure 22b shows the *m*-line spectrum of the other composite SiO_x_:TiO_y_ slab waveguide recorded using a grating coupler with the period *Λ* = 417 nm. The coupler was prepared by the nanoimprint method [16,29,30]. The resonant excitation angles of the mode TE_0_ are ±2.2924° and the excitation angles of the mode TM_0_ are ±5.6244°. Hence, the calculated from the formula [49,50,51,52]:

(19) $$N=n_{a}\sin\theta_{r}+r\frac{\lambda }{\Lambda }$$

effective refractive indexes are *N_TM_*_0_ = 1.52607 and *N_TE_*_0_ = 1.58408, while the refractive index and thickness calculated from the system of Equation (1) are, respectively, *n*_1_ = 1.7996 *d* = 186.4 nm. In the presented case, the diffraction order is *r* = 1.

Narrow peaks of the resonant excitation of the basic modes TE_0_, TM_0_ in both cases shown in Figure 22 bespeak of low optical losses in the waveguide layer, which is confirmed by the results of optical loss measurements presented below.

**Figure 23:**
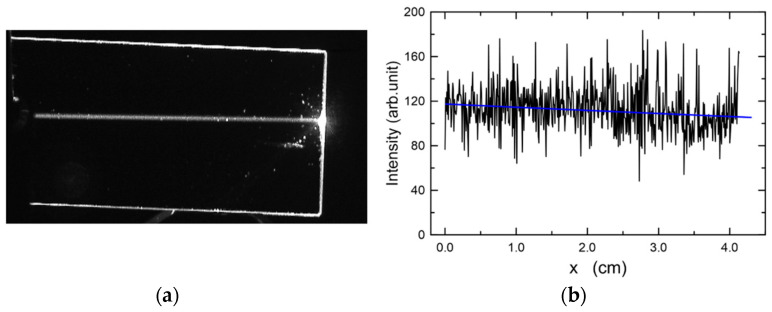
Scattered light streak accompanying propagation of a TM_0_ mode in a silica-titania slab waveguide on BK7 glass substrate (**a**) intensity distribution along the streak (**b**), *λ* = 677 nm, *d* = 181 nm, *n*_1_ = 1.8004.

**Figure 24:**
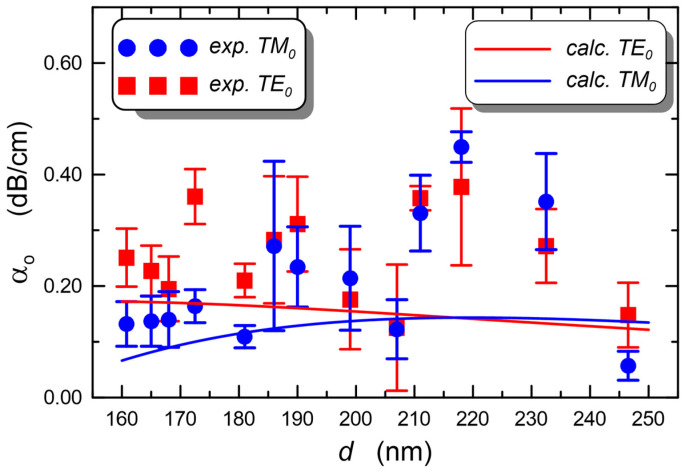
Influence of silica-titania layer thickness on optical losses of silica-titania waveguides on BK7 glass substrates, *λ* = 677 nm.

The composite slab waveguide SiO_x_:TiO_y_, in which a TM_0_ mode excited with a prism coupler is presented in Figure 23a. The prism coupler is not visible. This image was recorded in the measurement system shown in Figure 10. One can observe a streak of scattered light along the propagation path. The distribution of the intensity *I*(*x*) of the light along the streak is shown in Figure 23b. As one can observe, the intensity slightly decreases along the direction of propagation. The attenuation coefficient *μ*= 0.025(15) cm^−1^ and the optical losses *α* = 4.343 μ = 0.11(2) dB⋅cm^−1^ were determined from the approximation with the function *I*(*x*) = *I_0_*⋅exp(−*μx*). The latter is plotted with blue line. The same tests were performed for a series of layers made of the same sol, but at different speeds of substrate withdrawal *v*. Finally, all structures were simultaneously annealed at 500 °C for 60 min. Thus, the individual layers differed only in thicknesses. The experimental dependence of the optical losses *α*_0_ on the thickness *d* for this series of waveguide layers, for both basic modes TE_0_ and TM_0_, is shown in Figure 24. Within this thickness range *d*, each of the waveguide layers is single-mode (Figure 2). Blue filled circles were used to mark the losses of for TM_0_ modes, whereas red filled squares were used to mark the losses for TE_0_ modes. The lowest optical losses *α*_0_ = 0.06(3) dB⋅cm^−1^ were determined for the TM_0_ mode in the layer with a thickness of *d* = 246 nm (*α*_0_ = 0.15(6) dB⋅cm^−1^ for TE_0_), and the highest losses for the waveguide layer with a thickness of *d* = 218 nm, respectively *α*_0_ = 0.45(3) dB⋅cm^−1^ for the mode TM_0_ and *α*_0_ = 0.38(14) dB⋅cm^−1^ for the mode TE_0_. For seven waveguide layers, for the TM_0_ mode the optical losses are below 0.2 dB⋅cm^−1^, while for the TE_0_ mode, such a loss level was achieved for four waveguides. For the TM_0_ mode, the optical losses are greater than 0.3 dB⋅cm^−1^ in three cases, and for the TE_0_ mode in four cases.

The total scattering losses at the interfaces of the waveguide layer, calculated using the Lacey-Payne model, and plotted with solid lines are shown in Figure 24. In line with the thesis put forward in Section 3, we also used this model to calculate the losses for the mode TM_0_. The parameters of the surface of the waveguide layer and the surface of the BK7 substrate given in the caption of Figure 6 were adopted for the calculations. As one can see, the experimentally determined optical losses for both fundamental modes are similar to the calculated scattering losses with the use of the extended Lacey-Payne model. The experimental values of the optical loss *α_o_* are slightly higher than the calculated values. It may be affected by different values of surface roughness *σ* and autocorrelation length *L_c_* of the used substrates from those assumed for the calculations. The substrate glass plates were cut out from a glass block and then mechanically ground and polished. Indisputably, such a method of preparing the surface of the substrate, even despite great care, can result in a relatively large dispersion of the parameters of the substrate surface, i.e., its roughness *σ* and the autocorrelation path *L_c_*. Therefore, it is reasonable to suppose that different distances of the experimental points from the theoretical characteristics are the result of technological dispersion of surface parameters of the substrate glass (*σ*, *L_c_*). Surface quality of the substrate glass can be improved by introducing a buffer layer, which will smooth the surface of the substrate. Such a layer can be produced by the sol-gel method and dip-coating technique. By selecting the appropriate amount of titanium dioxide, it is possible to obtain a refractive index close to the refractive index of the substrate glass. Our first attempts in this area have yielded very promising results, which will be the subject of the next publication.

When we consider the relative relations of the measured losses *α_0_* for the modes TE_0_ and TM_0_ (Figure 24), it is easy to notice that despite their shift towards higher values, they accurately replicate the theoretical relationships. For smaller thicknesses *d* of the waveguide layer, the measured optical losses for the TE_0_ mode are greater than those for the TM_0_ mode, which is consistent with the calculation results. Next, as the thickness of the waveguide layers increases, this difference diminishes. For waveguide layers with the thicknesses above 200 nm, the experimentally determined optical losses are practically identical for both basic modes, as predicted by the extended Lacey-Payne model.

Based on the comparison of the experimental and computational results, we can state that the experimental dependences of optical losses on the thickness of the waveguide layer *α*_0_(*d*) confirm the correctness of the computational results for both TE_0_ mode and TM_0_ mode. Thus, the thesis put forward in Section 3 that the Lacey-Payne model can also be used for TM polarization has been proven here. At the same time, the observed compliance of the calculation results of the Lacey-Payne model with the experimental results indicates that the main source of optical losses in the presented optical waveguides are scattering losses on the boundary surfaces of the waveguide layer. The nature of the dependences involving scattering loss on nanocrystals contained in the waveguide layer (Figure 9) is different from the nature of the dependence involving scattering loss on the boundary surfaces. Thus, the compliance of the losses calculated from the Lacey-Payne model with the measured losses shows the dominant nature of the scattering losses on the boundary surfaces of the waveguide layer in the measured optical losses. Therefore, we can accept that the scattering losses on nanocrystals in the volume of the waveguide layer are within the measurement uncertainty.

**Figure 25:**
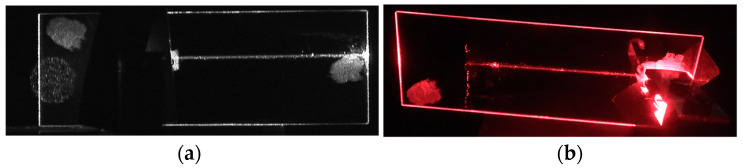
Scattered light streak from a silica-titania slab waveguide on BK7 glass substrate for the same waveguide as presented on Figure 20a, recorded for TM_0_ mode after 7 months (**a**), and after 13 years (**b**).

The series of waveguide layers on BK7 glass substrates for which the optical loss measurement results are presented in Figure 24 was produced in 2008. They were stored in Schiefferdecker staining dishes in room conditions, in an atmosphere that was not controlled. With the passing storage time of these waveguide layers, their parameters were controlled by ellipsometry measurements and by the assessment of propagation losses. We have already demonstrated that over the period of 5 years, the SiO_x_:TiO_y_ composite waveguide layers retained their properties, and optical losses remained unchanged [41]. Images of the same planar waveguide as the one in Figure 23a, excited in the set-up showed in Figure 10, are presented in Figure 25. The photo in Figure 25a was recorded 7 months after the photo in Figure 23a. The image presented in Figure 25b was registered 13 years later. As one can see, in each case a streak of scattered light is visible, the intensity of which in the direction of propagation is changing almost imperceptibly and the calculated losses are at the same level. These results prove the long-term stability of the parameters of the SiO_x_:TiO_y_ composite waveguide layers produced by the sol-gel method and dip-coating technique.

## 6. Conclusions

The paper presents the results of comprehensive tests of SiO_x_:TiO_y_ composite waveguide layers with high refractive index (*n*~1.8 at *λ* = 632.8 nm) for the application in the technology of planar evanescent wave sensors. The layers were produced with the sol-gel method and dip-coating technique on BK7 glass substrates. In the research the morphology of the waveguide layers and their optical properties were tested. The research with the use of atomic force microscopy (AFM) and scanning electron microscopy (SEM) yielded very high surface smoothness of the waveguide layer and much lower surface smoothness of glass substrate. Values of the surface roughness of bare substrate and composite waveguide layer surface were determined from areas of 10 × 10 μm^2^ using the AFM method. They are 1.03 nm and 0.29 nm, respectively. On the other hand, values the of autocorrelation length *L_c_* were determined from linear scans of length 1 μm. They are 11.4 nm and 72 nm for waveguide layer surface and substrate, respectively. The tests with the reflection spectrophotometry method demonstrated high optical homogeneity of the presented waveguide layers, and using the analysis of transmission spectra with the Tauc method, the optical band gaps for straight and oblique transitions were determined, whereof values are typical for amorphous structures. The amorphous nature of the material of SiO_x_:TiO_y_ composite waveguide layers was confirmed by the results of the HRTEM method. Owing to the amorphous nature of these layers, high smoothness of their surface is obtained.

Using the method of spectroscopic ellipsometry, dispersion characteristics of the refractive index *n* and that of the extinction coefficient *κ* were determined. Both ellipsometric spectroscopic measurements and spectrophotometric measurements demonstrated that the composite layers SiO_x_:TiO_y_ have good optical properties in the Vis-NIR spectral range. The test results with the *m*-line method showed that their propagation losses are low. Moreover, they indicated the impact of the thickness of the waveguide layer on the level of such losses, as well as the relationship between the losses for the modes TE_0_ and TM_0_. The analysis of the dependence of propagation losses on the thickness of the waveguide layer in combination with the results of theoretical analysis clearly showed that scattering at the interface waveguide layer/substrate is the main source of propagation losses, and it is affected by high roughness of glass surface and high value of the autocorrelation length. The impact of the surface quality of the substrate on the propagation losses is an order of magnitude higher than the impact of surface quality of the waveguide layer/cover. The lowest obtained propagation losses are at the level of 0.06 dB cm^−1^. The reduction of losses and the improvement of the quality of the waveguide layers can be achieved by smoothing the substrate surface through the use of a buffer layer. Such a layer can also be produced with the sol-gel method and dip-coating technique. Long-term studies of SiO_x_:TiO_y_ composite layers over a period of 13 years did not show any changes in their optical parameters. Thus, it has been demonstrated that the developed SiO_x_:TiO_y_ composite waveguide layers with high refractive index are stable over many years and are suitable for applications in the evanescent chemical/biochemical sensors technology.

## Figures and Tables

**Table 1:** Parameters of Tauc-Lorentz model fit to experimental data.

*E_g_*_1_ (eV)	*A*_1_ (eV)	*E*_01_ (eV)	*B*_1_ (eV)	*E_g_*_2_ (eV)	*A*_2_ (eV)	*E*_02_ (eV)	*B*_2_ (eV)
3.159 ± 0.004	38.0 ± 0.9	7.0 ± 0.2	14.0 ± 1.2	3.624 ± 0.003	68.5 ± 0.8	4.309 ± 0.005	1.755 ± 0.01

## Data Availability

Not applicable.
